# The effectiveness of specialized legal counsel and case management services for indigent offenders with mental illness

**DOI:** 10.1186/s40352-016-0038-6

**Published:** 2016-07-11

**Authors:** Jeff Bouffard, Elizabeth Berger, Gaylene S. Armstrong

**Affiliations:** 1 Department of Criminal Justice and Criminology, Sam Houston State University, PO Box 2296, Beto CJ Center, Huntsville, TX 77341 USA; 2 Police Executive Research Forum, 1120 Connecticut Ave. NW, Suite 930, Washington, DC 20036 USA

**Keywords:** Offenders with mental illness, Jail diversion, Indigent defense

## Abstract

**Background:**

In recent years, jurisdictions have recognized the strain placed on limited existing resources by criminal offenders with mental illness who frequently cycle through local jail facilities. In response, many locales have developed and implemented specialized programs to more effectively and efficiently manage these offenders, particularly the process of assigning defense attorneys to these often indigent defendants.

**Methods:**

The current study examined the impact of an Indigent Defense Counsel (IDC) program designed to provide specially trained defense attorneys, and enhanced case management services to 257 indigent jail inmates with a qualifying, major mental health diagnosis (e.g., major depression). These offenders were compared to 117 similar offenders who did not receive these services, on both their length of stay in the jail, and their likelihood of recidivism after release to the community.

**Results:**

Survival analyses revealed that program participants spent about 17 fewer days in jail; however, recidivism rates between groups, measured as return to the same county jail or as statewide re-arrest, did not differ.

**Conclusions:**

These results suggest that defendants with mental illness can potentially be managed effectively in the community, with little added risk to public safety and at potential savings in jail bed days/costs. Implications for the processing of indigent criminal defendants with mental illness are presented.

## Background

Individuals with mental illness are over-represented in the U.S. criminal justice system. In local jail facilities, they are estimated to comprise anywhere from 14 to 16 % of jail inmates (Steadman et al. 2009; Ditton 1999) to as much as 60 % of the inmate population (James and Glaze 2006, though the generalizability of this estimate has been questioned, see Slate et al. 2013). Although a daunting task, identifying individuals with mental illness and ensuring adequate service provision is crucial for preventing recurrence of violent offenses (Hiday et al. 2001), victimization (Hiday et al. 2001; Silver et al. 2005) and continued substance use (Swartz and Lurigio 2007). Research suggests that while mental illness may not be directly related to criminality, it can act as a mediator in the pathway to offending when coupled with other environmental or genetic risk factors. Some examples include prior victimization, pre-existing conduct or behavioral disorders, or substance abuse problems (Hiday et al. 2001; Hodgins et al. 2008; Swartz and Lurigio 2007).

Jails are often poorly equipped to address the needs of individuals with mental illness, which may both contribute to the high rate of recidivism among this population (e.g., 50.7 % vs. 38.7 % for those with no psychiatric disorder, Baillargeon et al. 2009; see also Blank Wilson et al. 2014) and exacerbate mental health problems stemming from incarceration (Beven 2005). According to James and Glaze (2006) jail inmates with mental illness are the least likely to receive treatment while incarcerated (17 %), followed by federal (24 %) and state prison inmates (34 %). When treatment is available in the jail, challenges are posed by the offender’s legal status, limited duration of detention, and unpredictable or unexpected release dates (Draine et al. 2010). Upon release, individuals with mental illness continue to exhibit high levels of treatment needs, such as indigence, difficulty finding housing, or substance use problems (Petrila and Skeem 2004; Gagliardi et al. 2004; Eastern Regional Conference of the Council of State Governments 2002; Epperson et al. 2011). Coupled with the high likelihood of indigence, it is not surprising that individuals with serious mental illness (i.e., Axis 1 disorders, like major depression or schizophrenia) have significantly higher rates of recidivism as compared to other groups (Baillargeon et al. 2009; Castillo and Fiftal Alarid 2010).

Some promising approaches to address the paucity of services available for individuals with mental illness include system-based approaches, such as specialized community supervision caseloads for mentally ill individuals in contact with the criminal justice system (Skeem et al. 2009; Burke and Keaton 2004) and more comprehensive and intensive programming, particularly mental health courts (Redlich et al. 2006; Ray et al. 2014; Sarteschi et al. 2011; Ray 2014; Moore and Hiday 2006; Hiday et al. 2013). As intensive interventions (i.e., mental health courts) can be expensive and resource-dependent, some jurisdictions have employed more limited responses, such as the appointment of public defenders who are trained specifically in mental health issues in hopes of better addressing the legal needs of these defendants (Pima County, Arizona, Mental Health Defender’s Office, 2016; Law Office of the Los Angeles County Public Defender, 2012). Whether attorneys, even those with specialized training, can provide adequate case management services (referring clients to needed treatment) is an open question however.

The current study presents findings from an innovative program designed to support indigent defendants who present in jail with one of three qualifying, major mental health diagnoses: major depression, bipolar disorder and/or schizophrenia. Individuals in the program being evaluated here were assigned a defense attorney who was specially-trained in mental health issues, and provided enhanced case management services including assessment and referral to various existing mental health and other social support services in the community, by licensed social workers employed by the Indigent Defense Counsel (IDC) program. Here, we examine the program’s impact on length of stay in jail and subsequent recidivism (return to this jail and re-arrest anywhere in the state), in comparison to a group of similar indigent, mentally ill inmates who were not provided a specially-trained defense attorney, nor did they receive enhanced case management services.

Not every jurisdiction may have the financial and organizational resources to field more comprehensive, and costly mental health court program. In light of this, it is important to consider whether more limited interventions such as this can yield positive results (e.g., no increase in recidivism) that would support the movement of mentally ill, indigent defendants out of jail while they await the disposition of their case. Doing so has a number of potential benefits, including reducing jail crowding and expenses related to housing mentally ill pretrial defendants, providing needed community-based treatment to these defendants, and breaking the cycle of repeated incarceration of defendants whose mental health issues go unremediated.

### Mental illness and criminality

Research shows that mental illness may act as a significant mediator in the pathway to offending when coupled with other environmental or genetic risk factors, such as prior victimization or pre-existing behavioral disorders (Hiday et al. 2001; Hodgins et al. 2008). A direct relationship between mental illness and criminal behavior can be observed in about ten percent of cases. In other instances though, issues such as poverty, substance abuse, and other risk factors influence this relationship (Peterson et al. 2010; Swartz and Lurigio 2007; Epperson et al. 2011).

In addition to mental health-related impairments, these offenders tend to possess other criminogenic risk factors due to social disadvantages such as poverty, increased law enforcement presence in their neighborhoods, unemployment, and other issues that exacerbate risk factors (Epperson et al. 2011). For similar reasons, the stigma toward people with serious mental illness exacerbates criminogenic risk, resulting in an increased likelihood of arrest for such individuals (Epperson et al. 2011), although Engel and Silver (2001) also found that controlling for a number of other factors (e.g., offense type, demeanor) mental health status was either unrelated to or actually reduced the likelihood of arrest. Beyond police decision making, policy decisions including the deinstitutionalization of state hospitals and holding persons awaiting movement to mental health facilities in jails (James and Glaze 2006) have further increased this proportion of individuals with mental illness housed in jails. Ineffective reentry processes and/or lack of sufficient community mental health programming can also reduce the chance that this population successfully reintegrates to the community, increasing their likelihood of returning to these same facilities (Ventura et al. 1998; Loveland and Boyle 2007; Epperson et al. 2011).

While incarcerated, offenders with mental illness experience greater difficulty adjusting to periods of incarceration, and frequently exhibit an inability to follow institutional rules and regulations. The result is a much higher likelihood that offenders with mental illness are charged with a rule violation, engage in physical or verbal assault while incarcerated in jail (James and Glaze 2006) or are placed in isolation, all of which can serve to further aggravate their mental health condition (Beven 2005). Overall, inmates with mental health problems may pose unique problems within correctional settings in comparison to other subpopulations.

### Responding to offenders with mental illness

A number of jurisdictions have developed procedures to channel mentally ill offenders from the justice system altogether, through use of various types diversion programs (Epperson et al. 2011). When diversion is not possible, the next option would be the immediate provision of screening and stabilization services upon arrest, continued treatment while incarcerated, and transition to appropriate and consistent treatment services in the community upon release (Swartz and Swanson 2002). Unfortunately, various barriers to this desired continuum of care exist, including the lack of resources needed in jails to perform accurate identification of mentally ill offenders, and to provide sufficient clinical services to the offender both while incarcerated, and again in the community after release.

Proper screening of detainees is crucial during the booking stage to prevent interruption of services while entering jail from the community (Veysey et al. 1997). Inadequate mental health screening upon booking into the jail is likely to exacerbate existing mental illnesses due to the abrupt discontinuation of treatment. Further, as part of post-release transition into the community, offenders with mental illness need an adequate discharge plan that incorporates community-based mental health treatment, social services, and housing to provide support for reintegration (Veysey et al. 1997). In addition to challenges regarding identification of offenders with mental illness, insufficient institutional capacity to provide a comprehensive range of services exists in most jails (Sung et al. 2010). Though approximately 731,000 inmates were held in over 2,800 American jails in 2013 (Minton and Golinelli 2014), many of these jails are very small (i.e., 639 jails have 25 or fewer beds) resulting in significant limitations in level and type of care afforded (American Jail Association 2003). When services are available, they tend to be limited such that a significant proportion of those in need of treatment are awaiting mental health services (Sung et al. 2010).

With the recognition of the limited services available in jails, policymakers and practitioners have explored various ways to respond to the needs of offenders with mental illness. Recent focus has been placed on improving the continuum of care, increasing access to appropriate services, and reducing the length of jail stay (Epperson et al. 2011). These approaches have included diversion to community-based outpatient programs or inpatient/residential facilities, pretrial release programs, and specialized probation caseload services (Hiday et al. 2013; Castillo and Fiftal Alarid 2010).

### Mental health courts

While representing possibly the most comprehensive and resource intensive responses, research has supported the use of mental health courts, especially in terms of their effects on reduced recidivism (McNiel and Binder 2007; Ferguson et al. 2008; Steadman et al. 2011; Hiday and Ray 2010) and substance abuse (Frailing 2010), as well as in promoting independent living and improving mental health functioning (Frailing 2010; Cosden et al. 2003; Cosden et al. 2005). Mental health court programs combine enhanced court involvement, intensive probation supervision, and community-based treatment into one model that serves as an alternative to incarceration. Assessments of the literature show that mental health courts are a promising practice, but more research is necessary to fully develop an evidence-based model (Edgely 2014; Honegger 2015). However, the complexity of the programs may place them outside the realm of possibility for some jurisdictions.

Although the goals of mental health courts generally tend to focus on improving quality of life for defendants and the community (via reductions in recidivism and improved mental health), program components and implementation can vary greatly. A typical mental health court utilizes a non-adversarial team approach where criminal justice and mental health stakeholders work together to develop individualized plans for mentally ill offenders. Often, mental health courts include treatment programs, meetings with mental health professionals, drug screenings and regular appearances in front of the judge (Ray et al. 2014). Within the various mental health court models, some programs employ specialized probation caseloads for mentally ill offenders (Lurigio et al. 2012; Castillo and Fiftal Alarid 2010). Larger-scale literature and quantitative reviews have deemed mental health courts as a promising practice, but due to the wide variation in implementation and methodological features of the evalulation such programs, the mental health court model itself has yet to reach the level of an ‘evidence-based model’ (Sarteschi et al. 2011; Honegger 2015).

Although mental health courts are still an evolving practice, a growing body of evidence supports their efficacy in reducing recidivism (Edgely 2014; Frailing 2010; Moore and Hiday 2006). A quantitative review done by Sarteschi et al. (2011) suggests several benefits of mental health courts, such as reduced recidivism, reductions in costs, and ‘decriminalization’ of the mentally ill population (Sarteschi et al. 2011). Sarteschi et al. (2011) quantified the average recidivism reduction from mental health courts, calculating a Cohen’s D effect size of −0.54 across several studies. An additional finding of Sarteschi et al. (2011) quantitative review was that success of a mental health court is related to the quality and multifaceted nature of services provided throughout the program. However, when a complex array of services is offered within a mental health court, it becomes a very resource-intensive option. Though mental health courts appear to be a promising practice, they may not be feasible to implement in all jurisdictions, particularly when resources are limited.

Short of full-fledged mental health courts, some jurisdictions have developed specialized probation units (SPUs) or specialized caseloads for mentally ill offenders that are smaller in size and have a primary focus of responding to mental health issues (Skeem et al. 2006; Castillo and Fiftal Alarid 2010). In contrast to a mental health court, SPUs are able to serve clients from *any* of the criminal court dockets (Morrissey et al. 2009). Probation officers in these units are specially trained in mental health problems and can appropriately respond to crisis situations (Castillo and Fiftal Alarid 2010; Council of State Governments/Eastern Regional Conference, and United States of America 2002). Although SPU program specifications vary widely, specialized caseloads that exclusively focus on mental health are generally more effective than traditional caseloads for mentally impaired offenders (Skeem et al. 2006).

Multiagency collaboration between state and local providers could improve the coordination of in-jail and community-based services for mentally ill defendants. Agency participants may include mental health professionals, substance abuse treatment providers, hospitals, housing providers, law enforcement, and representatives from state criminal justice, mental health, and substance abuse agencies (Conly 1999; Boothroyd et al. 2003). Due to the multi-faceted needs of mentally ill offenders, it stands to reason that models requiring collaboration between multiple agencies and therefore linkage to a greater variety of services would have the most promise for success (Baillargeon et al. 2009).

### Indigent defense services

Another challenge for offenders with mental illness is that these individuals are often indigent, and as a result cannot afford legal counsel. In some jurisdictions, resources exist to provide mentally ill offenders with legal counsel specially trained in mental health issues, but oftentimes these resources are not available (Mental Health Legal Advisors Committee 2014). For example, the Mental Health Legal Advisors Committee (MHLAC) of Massachusetts contains fourteen judges and lawyers who are experienced in mental health law and perform advocacy work to ensure that mentally disabled persons are protected under the law. MHLAC staff provide legal referrals, information, and advice to individuals, professionals and the general public; actively working to maintain and promote the administration of justice related to mentally ill offenders (Mental Health Legal Advisors Committee 2014).

While there is only emerging recognition of the need for indigent defense services for defendants with mentally illness, general indigent defense provisions are typically available across the country, though there is wide variation regarding models of service delivery and quality of services provided (Laurin 2015). Due in part to the lack of consistency in indigent defense policy, little is known regarding how these systems operate and what outcomes they intend to achieve (Laurin 2015; Tonry 2013). Research on indigent defense programs in general is still evolving, and many of the existing studies compare case outcomes of public defenders with privately assigned attorneys (Hartley et al. 2010; Williams 2013; Roach 2014), with no specific examination or measure of how these programs may be applied to those with mental illness. Development of evidence-based indigent defense practices is a growing priority for researchers and practitioners alike. In recent years, a handful of jurisdictions have prioritized their shift toward evidence-based indigent defense, as seen prominently in some states such as North Carolina, Texas, and New York (Laurin 2015).

North Carolina was one of the first states to adopt a data-driven mission, and gained an Office of Indigent Defense Services (IDS) in 2000. IDS focused on enhancing the quality and uniformity in indigent defense services, and prioritized adequate data entry and the production of reliable data as a core part of their mission (Laurin 2015; Gressens and Atkinson 2012). Studies suggested that North Carolina public defenders were not only more economical than private attorneys, but that they conducted more activities than the privately assigned attorneys. Activities of the public defenders were found to be programmatically important to improving the efficiency of the court system. Specifically, public defenders managed the attorney appointment system, represented indigent defense on committees and boards to improve overall efficiency of the system, and provided resources for new and current attorneys to improve overall quality of indigent defense (North Carolina Office of Indigent Defense Services 2008).

Similarly, Texas introduced the Texas Indigent Defense Commission (TIDC) in 2001 with the goal of developing standards for indigent defense practices, in addition to a research base to identify and guide the implementation of evidence based practices (Laurin 2015). The Mental Health Division was also created to provide specialized defense services for mentally ill misdemeanants. By comparing case management approaches and outcomes across groups (privately assigned attorneys vs. public defenders), some researchers have found public defenders to be more affordable per-case than the private counsel group (Fabelo et al. 2013; Carmichael and Marchbanks 2012). Carmichael and Marchbanks (2012) explain that public defenders provided more services and obtained more case dismissals, deferred sentences, and acquittals than the private counsel group (Fabelo et al. 2013). Researchers also found that clients with mental illness achieved better outcomes when working with a public defender as compared to a privately appointed attorney (Fabelo et al. 2013). Specifically, mentally ill clients working with specialized mental health public defenders had more case dismissals, fewer guilty verdicts and were more likely to receive probation when convicted, as compared to mentally ill clients who had privately assigned attorneys (Fabelo et al. 2013).

Though there has been an increase in data collection and empirical analysis of the efficacy of various indigent defense systems, there is still relatively little empirical evaluation on the impacts of indigent defense, especially in regards to services for those with mentally illness. Given the high likelihood of indigence among the mentally ill offender population, it is possible that court-based programs could be more effective if specially trained legal counsel were part of a broader program of support, however it is not clear that even attorneys trained in mental health issues have the skill or time to engage in adequate case management for their clients. As such, more rigorous evaluations of indigent defense programs are warranted, especially as applied to this population of defendants with mental illness.

### Current study

Court-based programs for mentally ill and/or indigent offenders are a relatively new practice. The primary goal for many of these new approaches is improved identification and assessment of those with mental illness, more efficient case management, enhanced quality of associated services, and reduction in subsequent recidivism levels. Unfortunately, little research exists on court-based models of support for mentally ill individuals (although other court programs, like drug courts have generated considerable research), and program evaluations are sparse, often yielding equivocal results. Quantitative reviews of the existing literature demonstrate the wide variation in both implementation and outcomes associated with programs for mentally ill offenders, and consistently emphasize the need for rigorous program evaluations of existing and future programs (Edgely 2014; Honegger 2015; Sarteschi et al. 2011).

As such, the current study is timely in that it examines the combined impact of legal counsel and additional case management services on length of jail stay and recidivism outcomes for mentally ill, indigent defendants. Importantly, this program is less resource-intensive than a formal mental health court and while those comprehensive programs may produce positive results, their costs and resource requirements may limit the ability of smaller jurisdictions to implement such programs. The current IDC intervention on the other hand, involves only a small number of attorneys and case workers, such that if the results show even modest effects it might provide a response to the issues posed by defendants with mental health issues in jails that is more feasible for some agencies. Specifically, this paper presents the results from an evaluation of a program targeting indigent jail inmates who are diagnosed with one of three serious mental illnesses and compares them to similar offenders who receive representation by regular indigent defense attorneys (with no specialized mental health training) and no case management services. Outcomes examined in this study include the length of time in jail, as well as several measures of recidivism, including return to the same county jail and re-arrest anywhere in the state.

## Method

### Program description

The broader IDC program identifies offenders in county jail who have had prior mental health treatment and if indigent, provides those individuals with the opportunity to participate in a program that includes the appointment of one of twelve indigent defense attorneys, who have received specialized training in mental health issues. Defendants who have one of three qualifying major mental health diagnoses (i.e., bipolar disorder, major depression, schizophrenia) also received the services of program caseworkers who provided screening and referral to relevant, existing community-based treatment and other social service programs. Indigent defendants with some other diagnosis can still be assigned a specially trained IDC attorney, but do not receive enhanced case management services from the program and these defendants are not included in the current evaluation. The treatment group (those with special attorneys and case management) was compared to a group of similar offenders, also with one of the three qualifying diagnoses, who did not participate in the program (i.e., received appointment of a regular defense attorney and no specialized case management services). The comparison group received the standard treatment for indigent offenders, which included assignment to a defense attorney who has not received specialized mental health training. Defense attorneys assigned to defendants in the comparison group volunteer their services and were compensated with a flat fee paid by the court. Specifically, we examine the impact of this program on the length of stay in the local jail, and the likelihood of recidivism after program participation.

The IDC program is located in a suburban county in a large southwestern state and provides services to only defendants deemed indigent by the local county court system’s indigent defense office, after considering the defendant’s net household income and assets (e.g., 125 % of the federal poverty level in terms of net household income). Defendants are screened for potential eligibility at the time of their booking into the local jail by jail staff who check a state-level database of records of those who have ever received mental health treatment. Individuals who do not appear in this database, but present at the jail with evidence of current mental health issues may also be referred to the program. IDC program caseworkers receive a list of all such potential participants from the booking officer each week day and then contact potential participants to screen them for mental illness and explain the program and give them the opportunity to participate. In terms of current offense types, all defendants except those charged with capital murder are eligible to participate in the IDC.

If the individual is diagnosed with major depression, bipolar disorder and/or schizophrenia they are eligible for enhanced case management services as part of the IDC program, and the appointment of a specially trained defense attorney. For those defendants who receive IDC case management services, the program social workers assess the person’s mental health needs and social functioning and attempt to match them to available clinical services that exist within the local community, as well as any other types of social services (e.g., housing, employment) that may be needed. If the caseworker determines that the individual has some other kind of mental health issue, the defendant can still partially participate in the program—that is, by being assigned a specially trained defense attorney, but they will not receive the enhanced case management services. These individuals are not included in this study as part of either the treatment or comparison groups because they differ from these groups in their mental health issue diagnoses, and are thus not comparable to either group examined here.

Defendants who do not choose to participate in the IDC program at all follow normal court procedures for the assignment of an indigent defense attorney (who is not part of the IDC program and has not received specialized training) and receive no enhanced case management services (no other program in this county’s court system offers such services). The current study thus examines those individuals appointed an IDC defense attorney, who had one of the three diagnoses that qualify them for the enhanced case management services (*n* = 257), relative to other indigent defendants who have one of these same three diagnoses, but chose not to participate in the IDC (*n* = 117; no case management, assigned a regular indigent defense attorney).

Those mentally ill, indigent offenders who have one of the three qualifying diagnoses receive the additional case management services from the program’s social work staff, who work with the appointed IDC defense attorney to promote community stabilization of the individual through referral to needed clinical and social services, compliance with any relevant conditions of release from the jail, and desistance from criminal activity. These social workers provided an average of 5.1 total referrals (e.g., mental health, housing, employment) per IDC participant, and 2.0 mental health service referrals specifically per IDC participant. In addition to the enhanced case management services, a group of twelve attorneys volunteered to be part of the IDC program, and each received several hours of specialized training in mental health issues (typically six hours per year) from the IDC program social work staff. For instance, these attorneys take part in training which focuses on the symptoms of mental illness, the impact of such symptoms on their client’s social functioning and legal culpability, and the role of these symptoms as potential mitigating factors. Training also covers the possible side effects of medications used to treat such mental illnesses and how those side effects may also impact criminal culpability.

Unlike a more comprehensive Mental Health Court model, the IDC program does not include any enhanced judicial supervision (they must appear in court for scheduled hearings as any defendant would). In addition, any conditions of pre-trial release are monitored only by a bail bonds person (if the defendant was released by a financially-secured bond, as opposed to release on personal recognizance), not by a probation or pretrial release (governmental) authority. Participants in our comparison sample, likewise do not receive any particular judicial or pretrial supervision, other than that they must also appear in court for relevant hearings and may be monitored by a bail bonds person if they were released on a financial bond. The IDC program aims to quickly remove eligible program participants from the jail environment, so that their mental health needs may be more effectively addressed in community-based programs. Participation in the IDC program does not guarantee early release from the jail, as that decision still remains with the presiding judge, including whether to allow a personal recognizance bond, to set a financial bond amount, and any other conditions of release. If they are released from the jail (about 87 % of IDC participants were), IDC program participants meet with a program caseworker for additional assessment of social and clinical service needs (e.g., housing, mental health), provision of relevant referrals for service, and regular meetings to monitor the individual’s progress while in the program. Because the IDC program includes several components (enhanced case management and specially trained defense attorneys) it is not possible to determine which component may account for any significant program effects relative to the comparison group, who received neither the case management, nor legal defense enhancements.

### Data sources

IDC program staff collected quantitative data related to IDC participants and the comparison group cases directly from various computerized county court record systems from January 1, 2012 through March 30, 2013. Information on defendant’s dates of entry and release from jail, their current offense date and type, prior criminality (e.g., number and type of prior adult arrests), as well as various demographic factors (e.g., age, gender, race, ethnicity) was collected. In addition, recidivism data was collected from the state’s criminal records system, reflecting the date and type of any re-arrests in the state after release from jail. Recidivism information was collected for a minimum of 12 months post-release from jail (time-at-risk in the community) for both the IDC and comparison group.

### Participants

During the 15-month evaluation period, the IDC program provided enhanced case management services and assigned specially trained defense attorneys for 257 indigent defendants who had a diagnosis of bipolar disorder, major depression, or schizophrenia-type disorders. This represents 68.7 % of those identified with a qualifying diagnosis over this time period. During the same time period, another 117 indigent individuals with similar diagnoses were booked in the local county jail; however, these individuals chose not to participate in the IDC program. While both groups are comprised of indigent defendants with similar mental illness diagnoses, those who volunteered to enter the IDC program may differ from those in the comparison group who chose not to participate (e.g., on their motivation to enter the program), thus, multivariate analyses were used to control for criminogenic factors that could be related to the individual’s decision to participate in the IDC program and their risk of recidivism, including criminal history and current offense type.

The IDC and comparison groups are largely similar in terms of age, racial/ethnic composition, and current criminal offense. Recall also that all participants in each sample had been classified by the court system as indigent, and had been diagnosed with a qualifying disorder (major depression, bipolar disorder or a schizophrenia disorder). The groups were generally similar in the distribution of these diagnoses as well. For instance, bipolar disorder was the largest proportion of each sample (56 % of the comparison group and 59.6 % of the IDC group, *χ* ^2^ = 0.419, n.s.), followed by major depression (20.0 % of the comparison group, 20.7 % of the IDC group, *χ* ^2^ = 0.024, n.s.) and schizophrenia (4.3 % of the comparison, 7.8 % of the IDC group; *χ* ^2^ = 1.583, n.s.). As indicated in Table 1, the average age of defendants in the IDC group is 33.4 years, and 35.0 years for defendants in the comparison group (t = 1.350, n.s.). The IDC participants and the comparison groups are primarily comprised of defendants who are White (86 and 85.5 %, respectively), with some participants reported to be of Hispanic ethnicity (8.6 and 6.8 %, respectively).

**Table 1:** Characteristics of the IDC and comparison samples

Variable	IDC	Comparison	Test statistic
Age	33.4	35.0	t = 1.350
% Male	51.4	61.5	*χ* ^2^ = 3.358†
% White	86.0	85.5	*χ* ^2^ = 0.018
% Hispanic	8.6	6.8	*χ* ^2^ = 0.323
% with Any Current Felony	55.3	65.8	*χ* ^2^ = 3.694†
% with Current Drug/Alcohol Offense	32.8	34.2	*χ* ^2^ = 0.069
% with Current Violent Offense	26.2	24.6	*χ* ^2^ = .107
% with Current Property Offense	19.1	20.2	*χ* ^2^ = 0.054
Average # of Prior Arrests	5.13	6.38	t = 2.023*
# of Prior Arrests per Year	.150	.177	t = 1.625

† *p* < .10, * *p* < .05

The comparison group had slightly more male defendants, and defendants with a felony charge compared to the IDC group, but these differences were not statistically significant. Both groups were equally likely to have defendants with a violent, property, or drug/alcohol-related offense (including DUI) as the most serious current charge. While the comparison group had about one more prior arrest (6.38 vs. 5.13, t = 2.023, *p* < .05) than did the IDC group, this difference appears to be a function of the somewhat higher average age of the comparison group. For instance, IDC participants had prior arrests at a rate of .150 per year, while comparison group participants prior arrest rate was .177 per year (t = 1.625, n.s.). Overall, despite the potential for self-selection bias in these samples, there seem to be few significant differences in a number of criminogenic factors between these two groups. Subsequent multivariate analyses, described below control for these factors.

### Outcome measures

Recidivism outcomes were measured in three ways: return to the local county jail for any reason, return to the jail for a new crime, and re-arrest anywhere in the state (i.e., search of the state police criminal records database) subsequent to release from the jail. Time-at-risk for each of these three variables was measured from the date of release from jail through either the date of first re-offense, or the end of the data collection period (when a 12-month check of their arrest records was conducted) if recidivism did not occur. Recall that recidivism outcomes were generated based on records showing that the person returned to the local county jail (for any reason and for a new crime, specifically), and a check of the state police department’s criminal record database, indicating the person had been re-arrested somewhere in the state. These comparisons include only those individuals in each group who had in fact been released from the local jail and thus has some period of time at risk in the community during which to potentially recidivate.

## Results

Results presented below include simple bivariate comparisons of each group’s likelihood of being released from the jail to demonstrate the possible benefit of the IDC program in helping to remove indigent, mentally ill offenders from the jail sooner than they would otherwise be released. In addition, we present bivariate comparisons of each of our three recidivism measures between the two groups, as well as a series of multivariate models for each of the three outcome measures. Specifically, a series of Cox regression survival analyses depict whether there are differences in the recidivism rates (e.g., return to the jail) of indigent, mentally ill offenders who received enhanced case management, and legal services (IDC group) compared to those who did not, while controlling for other factors such as offender demographics and offense information. These multivariate Cox regression models are “time-to-event” comparisons that determine the probability of recidivism, while controlling for time until that event, as well as the covariates just described (e.g., demographics).

### Release from the jail

Participation in the IDC program did not guarantee that the offender would be released from the jail (that remains at the discretion of the judge), yet a higher percentage of IDC program participants (87.2 %) were released from the jail during the study period than similar offenders who chose not to participate in the IDC (75.2 %, *χ* ^2^ = 8.296, *p* < .01; Cohen’s *d* = .30). Cohen’s (1988) rule of thumb for effects sizes (known as *d*) suggests that a *d* value over 0.2 would be considered at least a small effect, with a *d* over 0.5 being considered a moderate effect (Cohen 1988, pg. 25–26). Likewise, IDC program participants were significantly more likely to be released from the jail specifically by securing a financial bond (47.2 %) than comparison group participants (34.0 %, *χ* ^2^ = 5.096, *p* < .05, Cohen’s *d* = .23). While community release is not guaranteed as a part of IDC participation, and IDC staff have no direct influence on the release decisions of judges or bail bonds personnel, it is possible that these decision-makers perceive defendants who have agreed to engage in the IDC program to be more suitable candidates for release than defendants who chose to not participate in the program. For instance, participating in IDC means the defendants have an additional person (i.e., an IDC caseworker and specially trained attorney) involved in monitoring their status in the community (Table 2).

**Table 2:** Bivariate comparison of three recidivism outcomes between the two samples

Variable	IDC group	Comparison group	Test statistic	Effect size
Average Number of Days Spent in Jail	44.7	69.7	t = 2.77*	d = .31
% Returning to Jail after Release	31.6	28.2	*χ*2 = .405	d = .06
% Returning to Jail for New Charge	52.7	69.0	*χ*2 = 2.252	d = .16
% Re-arrested Post-Release (Statewide)	40.9	35.0	*χ*2 = 1.142	d = .11

* *p* < .05

Not only are IDC participants more likely to be released from the jail than those in the comparison group, but IDC participants are released more quickly (44.7 total days in jail) than those in the comparison group (69.7 days; t = 2.767, *p* < .01, Cohen’s *d* = .31). On average, this is a sizable and statistically significant difference of 25 days. The average cost to house an inmate with a diagnosed mental illness in a county jail in this state during the study period was $137 per day (per the state’s Legislative Budget Board). If the average number of days in jail for 257 IDC program participants is 25 days fewer than it otherwise would have been, the cost savings realized was approximately $880,000 over the course of the 15 months of this study.

### Recidivism outcomes

We next turn to an examination of the recidivism risk for IDC program participants as compared to similar offenders who chose not to participate in the program. Specifically, this section examines the impact of the additional indigent defense and case management services on recidivism for defendants with a diagnosis of bipolar disorder, major depression, or schizophrenia-related disorders (i.e., a “qualifying diagnosis”), relative to similar offenders who chose not to participate in the program. An examination of bivariate statistics indicates that IDC participants (31.6 %) were no more likely than their non-IDC counterparts (28.2 %, *χ* ^2^ = .405, n.s.; Cohen’s *d* = .06) to return to jail for any reason (i.e., new crime or revocation of bond conditions). In addition, among offenders who returned to the county’s jail, only 52.7 % of the IDC group returned due to a new criminal charge. Although, this rate of return was less that the comparison group (69 %), it was not a statistically significant difference (*χ* ^2^ = 2.252, n.s.; Cohen’s *d* = .16). In terms of statewide re-arrest rates, bivariate analysis showed no significant difference between the groups, although IDC participants were slightly higher than comparison group participants (40.9 versus 35.0 %, *χ* ^2^ = 1.142, n.s.; Cohen’s *d* = .11).

### Multivariate models

In light of some initial group differences (i.e., number of prior arrests, gender, and felony level offenders) and to account for varying times at risk (from release until re-arrest or the end of the follow-up period), a multivariate survival analysis (i.e., Cox regression) was utilized. This multivariate model includes controls for age, gender (male = 1), race (non-White = 1), Hispanic ethnicity (yes = 1), total number of prior arrests, current offense level (felony = 1), violent current offense (yes = 1), drug/alcohol-related current offense (yes = 1) and property current offense (yes = 1), number of days the individual had spent in jail prior to being released, as well as IDC program participation (IDC = 1).

The magnitude and significance of each predictor variables’ effect on the likelihood of re-incarceration and/or re-arrest is indicated by the likelihood coefficients shown in Table 3, also known as the hazard ratio. In this instance, the coefficient represents the magnitude of change in hazards (i.e., the likelihood that one recidivates), when there is a one unit increase in the predictor variable. Values greater than one indicate an increase in the odds of the outcome, while values less than one indicate a decrease in the odds of that outcome.

**Table 3:** Survival models for recidivism one-year post-release from jail

	Return to jail for any reason		Return to jail for new crime		Statewide Re-arrest	
	β (SE)	Exp(B)	β (SE)	Exp(B)	β (SE)	Exp(B)
IDC participation	−.116 (.226)	.890	.101 (.325)	1.107	.069 (.211)	1.072
Age	−.019 (.011)	.981†	−.008 (.018)	.992	−.056 (.012)	.946**
Male	−.708 (.223)	.493**	−.225 (.319)	.798	.023 (.196)	1.023
Race	−.726 (.347)	.484*	−.189 (.515)	.828	−.277 (.278)	.758
Hispanic	.623 (.348)	1.864†	−.472 (.528)	.624	−.107 (.376)	.898
Felony Charge	.504 (.226)	1.655*	−.858 (.313)	.424**	−.462 (.213)	.630*
Violent Offense	.561 (.303)	1.652†	−.092 (.398)	.912	−.169 (.273)	.844
Drug Offense	.015 (.284)	1.015	.027 (.392)	1.027	−.453 (.256)	.636†
Property Offense	.428 (.322)	1.535	−.331 (.478)	.718	.218 (.272)	1.244
Total Prior Arrests	.109 (.020)	1.116**	.085 (.029)	1.089**	.130 (.019)	1.139**
Days in Jail	−.013 (.004)	.987**	.005 (.005)	1.005	−.003 (.002)	.997

†*p* < .10, *p < .05, **p < .01

**Fig. 1:**
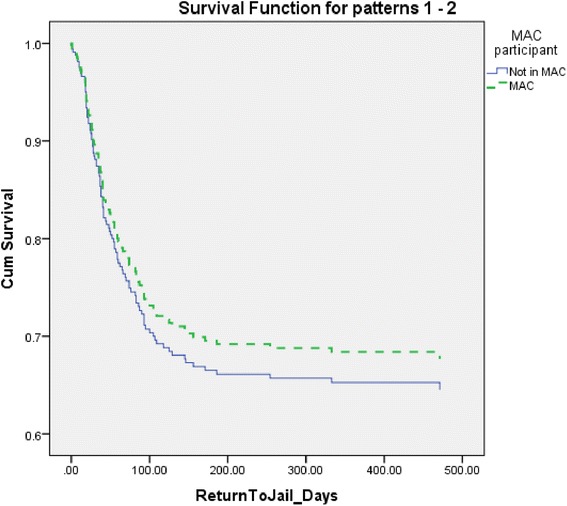
Survival Functions for Return to Jail (for any reason) by Groups

Results for the model predicting return to jail for *any reason* reveal that participation in the IDC program (β = −.116, S.E. = .226, n.s.) did not significantly impact the probability of this outcome (see Table 3). However, males had significantly lower probability of return to jail for any reason than females (β = −.708, S.E. = .223, *p* < .01), as did non-White relative to White defendants (β = −.726, S.E. = .347, *p* < .05); while felony level defendants (β = .504, S.E. = .226, *p* < .05) and those with more prior arrests had a significantly higher likelihood of return to jail (β = .109, S.E. = .020, *p* < .01). Finally, regardless of whether the individual was in the IDC or comparison sample, those who spent days in jail prior to release had a significantly lower probability of return to the jail for any reason (β = −.013, S.E. = .004, *p* < .01). Several other variables were marginally related to increased odds of return to jail for any reason, including Hispanic ethnicity and having been charged with a violent crime, while older individuals were marginally less likely to be re-incarcerated. Figure 1 depicts the survival curves for the MAC (dashed line) and comparison samples over time and generally reveal minimal differences in the rate at which each group survived (i.e., was not re-arrested; in consideration of space constraints figures depicting the other two recidivism measures are not presented though the patterns are similar).

In the model examining return to the jail for a *new crime*, IDC program participation was again not a statistically significant predictor of this outcome (β = .101, S.E. = .325, n.s.). Defendants facing a felony charge were less likely to return to jail for a new crime (β = −.858, S.E. = .313, *p* < .01), while those with higher numbers of prior arrests were more likely to return to jail for a new crime (β = .085, S.E. = .029, *p* < .01). No other variables were found to be significant predictors of the probability of returning to this particular jail for a new criminal charge.

Finally, in focusing on re-arrest anywhere in the state (data from the state police criminal record system), IDC program participation did not affect this outcome (β = .069, S.E. = .211, n.s.). The likelihood of re-arrest was significantly higher for offenders with a greater number of prior arrests (β = .130, S.E. = .019, *p* < .01), and was significantly lower for those with current felony offenses (β = −.462, S.E. = .213, *p* < .05) and older offenders (β = −.056 S.E. = .012, *p* < .01). Offenders charged with a current drug crime were also marginally less likely to be re-arrested (see Table 3).

While significant reductions in the likelihood of these recidivism measures among IDC participants was a program goal, which was not met, it is important to note that IDC offenders were also no *more likely* to recidivate when compared to their non-IDC counterparts. In other words, after controlling for all other factors, the odds of an IDC participant recidivating (measured with three different outcome variables) were not significantly different from those who did not receive the program’s services. That said, these IDC participants were released from the local jail nearly one month sooner than similar indigent offenders with mental illness who did not enroll in the program, and yet they appear to have posed no increased risk to public safety because of it. While the data do not support the conclusion that IDC participation *improved* recidivism risk, they do suggest that early release of these kinds of indigent, mentally defendants, coupled with enhanced case management and legal services can generate some positive outcomes (i.e., avoided costs) for the local jail, and county without posing an additional public safety risk.

## Discussion

Specialized services for the mentally ill criminal population have emerged over time as policymakers continually explore ways to respond to the needs of this population. Recent efforts have focused on improving timely and accurate clinical assessments, matching of appropriate services with treatment needs, increasing access to appropriate services, and improving the continuum of care, while attempting to maintain public safety and offender accountability. Many jurisdictions have implemented specialized mental health courts, which typically combine treatment and surveillance components, to allow for the delivery of treatment services in a community setting (Almquist and Dodd 2009), and these comprehensive programs tend to be more effective than traditional court supervision for offenders with mental illness (Latessa and Lowenkamp 2005). The resources required to implement a comprehensive mental health court program however may be beyond many jurisdictions. This IDC program is not as resource-intensive as a formal Mental Health Court program, specifically in that it lacks mandated mental health treatment and enhanced community supervision. While these missing program components may account for some of the lack of significant beneficial program effects, at the same time there are some promising outcomes from even this limited intervention for indigent, mentally ill jail inmates. For instance, while IDC participation did not formally *guarantee* early release from jail as a program component, not only did this study find IDC participants were significantly more likely to be released from the jail, they were released on average 25 days earlier. IDC participants were also more likely than the comparison group to be released on a bond (47.2 % and 34.0 %, respectively). Given that judicial discretion and bond requirements were retained, it can be inferred that criminal justice decision-makers may view IDC program participants as more viable candidates for early release from jail.

Despite the earlier release of IDC participants, no difference in recidivism risk was apparent. Specifically, analyses revealed that individuals in the IDC group were no more likely than the comparison group to return to jail for any reason (i.e. new criminal charge or revocation of bond), nor to be re-arrested elsewhere in the state. Thus, while the IDC program decreased the average length of jail stay and associated costs, and facilitated the ability of indigent offenders with mental illness to retain appropriate community based treatment services this did not reduce re-offense risk. On the positive side, these individuals were not found to pose an *increased* risk to public safety for the community either.

Programmatically, it is important to note that this IDC program lacked a number of the components that would typically be found in a Mental Health Court program, most notably mandated mental health treatment participation, on-going judicial interactions, and enhanced community supervision. Mental Health Court program evaluations have demonstrated significant *reductions* in recidivism, whereas this IDC program did not. Thus, it may be that while this sort of less intensive IDC program could help reduce jail crowding and promote access to needed services in the community, the additional supervision and coerced treatment aspects of the Mental Health court model are needed to produce more substantial improvements in recidivism.

### Limitations

In terms of the research design itself, limitations of the current study include the use of a quasi-experimental design, and in particular a lack of controls for self-selection into the IDC. While our multivariate models include a number of important control variables, it is not possible to rule out potential self-selection effects (especially differences in motivation for treatment, as IDC participants volunteered to enter the program). It may be for instance that individuals who volunteered for the IDC were also better able to effectively present themselves to the judge/bonds-person at the time of pretrial release decision-making, and it was this fact, rather than IDC participation per se that accounted for higher rates of release for IDC participants. While the IDC and comparison groups were largely similar in terms of the proportions exhibiting each of the qualifying diagnoses for the IDC program (e.g., Bipolar disorder), data was not available on the severity of symptoms experienced by each defendant, for instance. Had this data been available it might have been possible to determine the severity of each individual’s mental illness at the time of incarceration and better control for such potential confounds. At the same time, we believe the inclusion of important criminogenic control variables in the multivariate analysis should have helped to reduce this potential bias. Likewise, individuals in this study were followed for only one year after program participation, and only in terms of their recidivism outcomes. It is possible that the lack of significant differences in recidivism risk could eventually emerge over the longer-term, and that the program had beneficial effects in other areas, such as obtaining more stable housing, improved employment, or better psychological functioning, which were not measured in the current study.

Finally, no information was available on the types and amounts of services that individuals in the comparison group may have received. While data on the number and type of clinical and other social services that IDC participants were referred to was available to the researchers, similar data on the comparison groups was not available specifically because they did not participate in any known programming that would have made (and recorded) such referrals. At the same time, if these defendants did receive types and levels of services that were comparable to those provided to IDC participants, any potential differences in recidivism outcomes between the two groups would be moderated.

## Conclusion

It is possible that a singular and limited approach to the complex mental health and social service needs of this population is not enough. Other researchers have noted that effective treatment should be supplemented with integrated services that address a range of psychosocial factors, as many of these offenders face a challenging array of problems (Edgely 2014). Further research is warranted to understand the complexities underlying mental illness and offending and improve case management services and the linking of offenders to appropriate community mental health resources, as well as ancillary support services such as housing and employment.

The IDC program was successful at selecting appropriate, amenable candidates for enhanced case management and legal services. Most of these individuals were then granted early release from jail and generally had no increased risk of recidivating at the one-year follow-up. These results suggest that with some minimal case-management service provision and attorney training, it is possible to manage this kind of offender in the community, with little apparent increased risk to public safety. In addition, the program potentially generated cost savings for the local jail, or at least allowed for the reallocation of beds that would normally be used to house defendants with mental illness for use with other, higher risk offenders. In jurisdictions where the implementation of a full-fledged Mental Health Court program is not possible, for practical or political reasons, the implementation of similar IDC programs targeting defendants with mental illness might be a useful step in dealing with the many problems these individuals pose for local court and correctional systems, and for improving their own quality of life by providing needed services in a less restrictive and detrimental environment than that of the local jail.
